# Insight into the antifungal mechanism of *Neosartorya fischeri* antifungal protein

**DOI:** 10.1007/s13238-015-0167-z

**Published:** 2015-05-22

**Authors:** Máté Virágh, Annamária Marton, Csaba Vizler, Liliána Tóth, Csaba Vágvölgyi, Florentine Marx, László Galgóczy

**Affiliations:** Department of Microbiology, Faculty of Science and Informatics, University of Szeged, Szeged, Hungary; Institute of Biochemistry, Biological Research Centre, Hungarian Academy of Sciences, Szeged, Hungary; Division of Molecular Biology, Biocenter, Innsbruck Medical University, Innsbruck, Austria

**Keywords:** *Neosartorya fischeri* antifungal protein, *Aspergillus nidulans*, cAMP/Pka signalling, Pkc/Mpk signalling, antifungal mechanism

## Abstract

Small, cysteine-rich, highly stable antifungal proteins secreted by filamentous Ascomycetes have great potential for the development of novel antifungal strategies. However, their practical application is still limited due to their not fully clarified mode of action. The aim of this work was to provide a deep insight into the antifungal mechanism of *Neosartorya fischeri* antifungal protein (NFAP), a novel representative of this protein group. Within a short exposure time to NFAP, reduced cellular metabolism, apoptosis induction, changes in the actin distribution and chitin deposition at the hyphal tip were observed in NFAP-sensitive *Aspergillus nidulans.* NFAP did show neither a direct membrane disrupting-effect nor uptake by endocytosis. Investigation of *A. nidulans* signalling mutants revealed that NFAP activates the cAMP/protein kinase A pathway via G-protein signalling which leads to apoptosis and inhibition of polar growth. In contrast, NFAP does not have any influence on the cell wall integrity pathway, but an unknown cell wall integrity pathway-independent mitogen activated protein kinase A-activated target is assumed to be involved in the cell death induction. Taken together, it was concluded that NFAP shows similarities, but also differences in its mode of antifungal action compared to two most investigated NFAP-related proteins from *Aspergillus giganteus* and *Penicillium chrysogenum*.

**Electronic supplementary material:**

The online version of this article (doi:10.1007/s13238-015-0167-z) contains supplementary material, which is available to authorized users.

## INTRODUCTION

The increased incidence of fungal infections and the fast development of drug resistant filamentous fungi causing mycoses, plant infections or damage to cultural heritages strongly demand for the development of new antifungal strategies. Antifungal peptides have great potential in these fields, and also have significant commercial potential on the global market for antifungals set to be worth $12.2 billion by 2016 (Montesinos, 2007; Duncan and O’Neil, 2013).

In this respect, cysteine-rich antifungal proteins secreted by filamentous Ascomycetes are promising candidates. The *Neosartorya fischeri* antifungal protein (NFAP) secreted by *N. fischeri* NRRL181 is a novel representative of this protein group (Kovács et al., 2011). NFAP effectively inhibits the growth of numerous filamentous Ascomycetes including potential human and plant pathogens (Kovács et al., 2011; Virágh et al., 2014). In our previous work we demonstrated that NFAP interferes with the organization of the cell wall, destroys the chitin filaments and triggers apoptotic/necrotic events through reactive oxygen species accumulation in the NFAP-sensitive *Aspergillus nidulans* (Galgóczy et al., 2013). We also reported that *Pichia pastoris* KM71H produces heterologous NFAP in an antifungally active and folded state, and the antifungal effect is comparable to the native protein (Virágh et al., 2014). In spite of these promising results, a detailed insight into the antifungal mechanism of NFAP is still missing. This, however, represents essential prerequisite for the practical application of NFAP. Therefore, the antifungal mechanism of NFAP on *A. nidulans* was examined in more detail in the present study.

The following objectives were addressed in this work: investigation of (i) the cell viability and apoptotic/necrotic processes, (ii) the membrane permeabilization activity, and (iii) the actin and chitin distribution in the presence of NFAP. Furthermore, we examined the (iv) the localization of NFAP; and (v) the signalling pathways involved in the mode of action of NFAP.

## RESULTS

### Cell viability and apoptotic/necrotic events in the presence of NFAP

**Figure 1:**
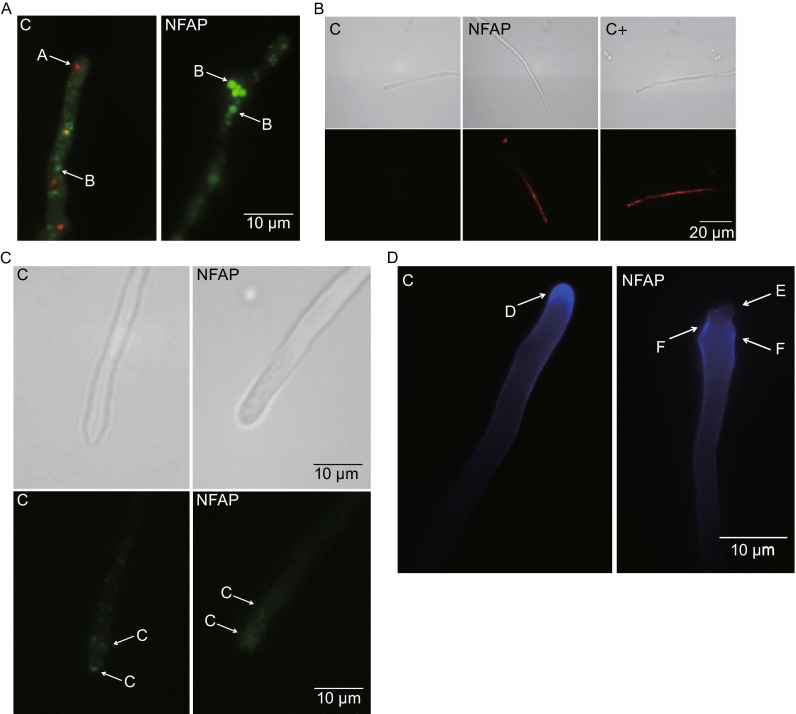
**Physiological changes in** ***Aspergillus nidulans*** **in the presence of** ***Neosartorya fischeri*** **antifungal protein (NFAP)**. (A) Viability staining of *Aspergillus nidulans* FGSC A4 hyphae with FUN-1 dye after NFAP treatment for 30 min at 37°C. Red vacuoles A indicates metabolic activity, while green vacuoles B metabolic inactivity. (B) Propidium iodide (PI) staining of *A. nidulans* FGSC A4 hyphae after NFAP treatment for 16 h at 37°C. Intracellular red fluorescence indicates membrane disruption. (C) Actin distribution at *A. nidulans* Actin-GFP hyphal tips in response to NFAP treatment for 30 min at 30°C. C: actin patch. (D) Calcofluor white (CFW) staining of *A. nidulans* FGSC A4 hyphae after NFAP treatment for 30 min at 37°C. D: cap-like CFW fluorescence - site of the chitin assembly, E: lack of the cap-like CFW fluorescence, F: delocalized chitin deposition. C: untreated control, C + : positive PI staining control-hypha was treated with 70% Et-OH for 1 h at 4°C, NFAP: NFAP-treated (25 µg/mL) hyphae. Upper images, light microscopy; lower images, fluorescence microscopy of PI staining (B) and of actin distribution (C)

The two-colour fluorescent FUN1 stain passively diffuses into the fungal cells and stains the cytoplasm and metabolically inactive vacuoles green, while the metabolically active vacuoles red. After short-time exposure to NFAP (30 min) reduced cellular metabolism was detected in *A. nidulans* FGSC A4 hyphae indicated by the presence of only green fluorescent vacuoles compared to the untreated control, which contained red fluorescent vacuoles too (Fig. 1A). This phenomenon was also observed after 60 min and 16 h of NFAP-treatment (data not shown).

Annexin V-FITC Apoptosis Detection Kit dyes the apoptotic cells green, while the necrotic cells are counterstained red. Living cells do not show any fluorescence. After 30 and 60 min a significant increase in the number of apoptotic 16 h-old germlings was observed in the NFAP-treated samples (26.1 ± 7.5% and 31.8 ± 8.8%, respectively; *P* < 0.0001) compared the untreated control where only a few percent of the cells (2.2 ± 1.3%) showed green fluorescence. Only few necrotic cells were detected in the untreated (5.1 ± 1.9%) and NFAP-treated samples after 30 min (4.8 ± 3.7%) and 60 min (5.2 ± 0.9%). These results suggest that NFAP exerts its antifungal effect through induction of apoptosis. After 16 h, however, almost all germlings (98.9 ± 1.1%) showed red fluorescence which indicated the death of the cells.

### NFAP does not cause direct membrane disruption

The membrane disrupting activity of NFAP was investigated by applying the membrane impermeant, red-fluorescent nuclear and chromosome stain propidium iodide (PI). This dye can penetrate only into the dead cells where the membrane integrity is damaged. Most of the *A. nidulans* FGSC A4 hyphae did not show any red fluorescence after a 30- and 60-min-long incubation in the presence of NFAP (data not shown). This indicates that the plasma membrane was still intact at these time points, similarly to the untreated control. After 16 h almost only red fluorescent hyphae appeared in the NFAP-treated sample pointing towards a massive membrane disruption as a consequence of the long-time cell killing effect of NFAP (Fig. 1B).

### Changes in the actin distribution and chitin deposition at the hyphal tip

To study the morphological aberrations of NFAP-exposed *A. nidulans* hyphae we analysed the actin distribution and the deposition of chitin at the hyphal tips. Actin-GFP expressing *A. nidulans* showed typical actin patch distribution at the hyphal tips clustered near the apical region and scattered behind the tips (Taheri-Talesh et al., 2008) (Fig. 1C). In contrast, actin patches were disturbed in hyphae that were treated with NFAP for 30 min only (Fig. 1C). Calcofluor white (CFW) staining revealed delocalized chitin deposition at hyphal tips of *A. nidulans* FGSC A4 after NFAP treatment for 30 min. In contrast, the untreated control sample exhibited a characteristic cap-like CFW fluorescence (Fig. 1D). These effects in actin and chitin delocalization were also observed after 60 min and 16 h of incubation with NFAP (data not shown). These results indicate that in the presence of NFAP the normal actin polarization/localization and chitin distribution are disturbed in *A. nidulans*.

### NFAP shows no active internalization

**Figure 2:**
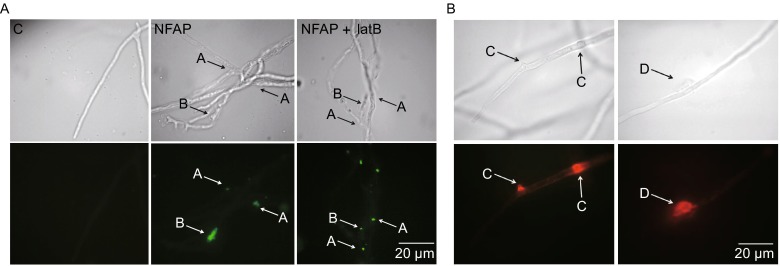
**Localization of** ***Neosartorya fischeri*** **antifungal protein (NFAP) in** ***Aspergillus nidulans***. (A) Indirect immunofluorescence staining of *Aspergillus nidulans* FGSC A4 hyphae with rat anti-NFAP serum and FITC-conjugated swine anti-rat IgG, after NFAP treatment for 16 h at 37°C. NFAP accumulation in A: hyphal fracture and twist; B: cell-wall bubble. C: untreated control, NFAP: NFAP-treated (25 µg/mL), NFAP + latB: NFAP- (25 µg/mL) and latrunculin B- (5 µg/mL) treated hyphae. (B) Propidium iodide (PI) staining of *Aspergillus nidulans* FGSC A4 hyphae after 25 µg/mL NFAP treatment for 90 min at 37°C. Red fluorescence at hyphal bubble C and around hyphal fracture D indicates membrane disruption and outflow of the hyphal/cell content. Upper images, light microscopy; lower images, fluorescence microscopy of indirect immunofluorescence staining (A) and of PI staining (B)

A produced polyclonal NFAP-antiserum reacted specifically with NFAP, and no signals were obtained with the control serum that was collected before the first injection (data not shown). An indirect immunofluorescence staining method was applied to study the localization of NFAP in *A. nidulans* FGSC A4. NFAP did not enter the fungal cell in detectable concentration levels after 30 and 60 min of exposure (data not shown), but after 16 h NFAP-specific fluorescence signals accumulated at hyphal fractures, twists and in cell-wall bubbles (Fig. 2A). To clarify whether the NFAP internalization was the consequence of an endocytic mechanism, the indirect immunofluorescence staining was repeated in the presence of 5 µg/mL latrunculin B (latB). LatB selectively inhibits the actin polymerization and therefore disturbs endocytosis. If the NFAP accumulation in the hyphae after 16 h is a consequence of an endocytotic mechanism no fluorescence signal should be observable in the cell. NFAP-specific fluorescence signals appeared in hyphae only after 16 h of incubation with NFAP and latB, but not after the exposure for 30 or 60 min (Fig. 2A). Based on these results, we conclude that NFAP is presumably not internalized by endocytosis. Instead, the accumulation of NFAP after 16 h is possibly a consequence of a passive diffusion at disrupted sites of the cell wall and plasma membrane.

### NFAP interferes with the G-protein signal transduction and cell wall integrity pathways in *Aspergillus nidulans*

#### NFAP activates the cAMP/Pka signalling cascade through heterotrimeric G-protein

The *A. nidulans* FGSC1035 strain carries a dominant negative mutation in the guanidine nucleotide binding domain of heterotrimeric G-protein α-subunit (*fadA*). As a consequence of this mutation the ability of the G_α_ subunit to dissociate from G_βγ_ is inhibited causing constitutive inactivation of heterotrimeric G-protein signalling (Yu et al., 1996). Growth inhibition assays revealed that FGSC1035 strain was less susceptible to NFAP than its isogenic recipient FGSC116 strain indicating that G-protein signalling is necessary for NFAP toxicity (Table 1). In *A. nidulans* the cAMP/protein kinase A (Pka) pathway is activated by heterotrimeric G-protein signalling, and it is involved in the regulation of morphological development (Shimizu and Keller, 2001), and in the induction of apoptosis (Semighini et al., 2006). To investigate if cAMP/Pka signalling plays a role in the mechanism of action of NFAP, we tested the susceptibility of an *A. nidulans* strain with depleted Pka activity (Shimizu and Keller, 2001). The *pkaA* deletion mutant exhibited reduced sensitivity towards NFAP compared to its recipient RKIS1 strain suggesting that NFAP activates the cAMP/Pka signalling cascade and possibly promotes programmed cell death (PCD) (Table 1). Our assumption was further supported by susceptibility tests applying NFAP in combination with 8-bromoadenosine 3’,5’-cyclic monophosphate (8-Br-cAMP) and caffeine on strain FGSC A4. 8-Br-cAMP is an activator of cAMP/Pka signalling in fungi (Gorovits and Yarden, 2003), while caffeine represses the Pka signalling by reduction of cAMP levels (Kuranda et al., 2006). NFAP aggravated the toxicity of 8-Br-cAMP indicating a synergistic interaction between the two compounds and an activating effect of NFAP in cAMP/Pka signalling (Table 2). In contrast, caffeine ameliorated the growth of FGSC A4 strain exposed to NFAP (Table 2). The *∆pkaA* strain, however, showed hypersensitivity (63% ± 6.5% growth) to 20 mmol/L caffeine compared to its recipient RKIS1 strain (91% ± 6.8% growth). This growth reduction induced by caffeine was cured by the addition of NFAP and overgrowth was detected compared to the untreated control (Table 2). These results suggest that NFAP and caffeine act in an opposite manner on Pka signalling. The overgrowth effect may be explained by the response of the fungus that activates rescue mechanisms against the co-administration of two antifungals, which have different cellular targets (Ouedraogo et al., 2011).

**Table 1:** Growth percentages of the investigated *Aspergillus nidulans* strains in presence of different concentrations of NFAP in *in vitro* broth microdilution test after 48 h of incubation at 30°C or 37°C (depending on the investigated strain)

NFAP/Strain	200 µg/mL	100 µg/mL	50 µg/mL	Type
RhoA^G14V^	48 % ± 15.2%^ns^	57% ± 4.5%^ns^	86% ± 12.5%^ns^	Mutant
*∆mpkA*	108% ± 9.1%***	105% ± 6.7%***	103% ± 0.9%**	Mutant
GR5	47% ± 7.5%	58% ± 5.4%	83% ± 11.9%	Isogenic recipient of RhoAG^14V^ and *∆mpkA*
*alcA*-PkcA^a^	48% ± 10.6%***	57% ± 0.6%***	64% ± 12.5%***	Mutant
*alcA*-PkcA^b^	74% ± 7.6%^ns^	77% ± 0.5%^ns^	99% ± 0.2%^ns^	Mutant
R153	72% ± 2.8%	83% ± 4.4%	102% ± 1.8%	Isogenic recipient of *alcA*-Pkca
*∆pkaA*	56% ± 6.1%***	69% ± 5.3%***	83% ± 11.5%**	Mutant
RKIS 1	40% ± 2.7%	48% ± 1.6%	66% ± 4.0%	Isogenic recipient of *∆pkaA*
FGSC 1035	52% ± 1.9%*	78% ± 0.2%***	101% ± 0.4%***	Mutant
FGSC 116	42% ± 6.7%	59% ± 1.9%	75% ± 2.3%	Isogenic recipient of FGSC 1035

The untreated control is taken as 100% of growth. The mean growth percentages and their standard deviations from three replicates (*n* = 3) are indicated in the cells. Significant differences (*P*-values) were determined based on the comparison with the growth percentages of parental strain. ***: *P* < 0.0001; ^**^: *P* < 0.005; ^*^: *P* < 0.05; ^ns^: no significant differences. NFAP: *Neosartorya fischeri* antifungal protein

^a^ *A. nidulans* *alcA*-PkcA in the presence of glucose

^b^ *A. nidulans* *alcA*-PkcA in the presence of glycerol

**Table 2:** Growth percentages of *Aspergillus nidulans* strains in presence of NFAP and NFAP-8 Br-cAMP/caffeine combinations in *in vitro* broth microdilution test after 48 h of incubation at 37°C

NFAP/other compounds	0 µg/mL	50 µg/mL	100 µg/mL	200 µg/mL
FGSC A4				
NFAP	100%	91% ± 6.0%	67% ± 6.7%	52% ± 3.2%
NFAP + 5 mmol/L 8-Br-cAMP	70% ± 1.6%	58% ± 4.2%***	57% ± 3.8%***	41% ± 3.2%***
NFAP + 20 mmol/L caffeine	71% ± 2.1%	74% ± 3.7%^ns^	61% ± 2.6%***	108% ± 1.5%***
*∆pkaA*				
NFAP	100%	83% ± 11.5%	69% ± 5.3%	56% ± 6.1%
NFAP + 20 mmol/L caffeine	63% ± 6.5%	123% ± 7.9%***	195% ± 30.6%***	196% ± 3.0%***

The untreated control is taken as 100% of growth. The mean growth percentages and their standard deviations from three replicates (*n* = 3) are indicated in the cells. Significant differences (*P*-values) were determined based on the comparison with the growth percentages in the presence of 8-Br-cAMP or caffeine alone. ***: *P* < 0.0001; ^ns^: no significant differences. 8-Br-cAMP: 8-bromoadenosine 3’,5’-cyclic monophosphate, NFAP: *Neosartorya fischeri* antifungal protein

It is worth to mention here that FGSC A4 and Actin-GFP strains showed the same changes in chitin deposition and the actin distribution after 60 min of exposure to 5 mmol/L 8-Br-cAMP (Fig S1a and S1b) as it was observed in the presence of NFAP (Fig. 1D and 1C). In contrast to the FGSC A4 strain (Fig. 1D), chitin delocalization was not observed in the *fadA* and *ΔpkaA* mutants when they were treated with 25 µg/mL NFAP for 30 min (Fig S2a and S2b). These observations further strengthen the role of the NFAP in the inhibition of polar growth and chitin assembly via cAMP/Pka signalling.

#### NFAP acts on an unknown cell wall integrity pathway-independent mitogen-activated protein kinase A-downstream target

RhoA is an essential protein for polar growth, branching, and cell wall synthesis in *A. nidulans* (Guest et al., 2004). The RhoA^G14V^ mutant with ectopic copies of the constitutively active *rhoA*^*G14V*^ allele showed the same susceptibility to NFAP and to the cell wall stressing agent CFW as its isogenic control GR5 strain (Table 1 and Table 3), but proved to be less susceptible to caffeine (Table 3). These results demonstrate that RhoA is not directly involved in NFAP toxicity, and NFAP may target the downstream effectors of RhoA, e.g. protein kinase C (Pkc). Pkc plays an important role in suppression of apoptosis through activation of the mitogen-activated protein kinase (Mpk) cascade, and also in polarity establishment independently of the Mpk cascade during germination in *A. nidulans* (Katayama et al., 2012). To investigate a possible role of Pkc/Mpk signalling in NFAP antifungal activity, we tested a conditional *A. nidulansalcA*-PkcA mutant for NFAP susceptibility. In this mutant, the expression of PkcA was repressed by glucose, but induced by glycerol (Ronen et al., 2007). There was no significant difference (*P* > 0.05) between the growth of this strain at different NFAP concentrations under PkcA repressing conditions in contrast to the isogenic recipient *A. nidulans* R153 (*P* < 0.0001) (Table 1). In the presence of glycerol the *alcA*-PkcA mutant showed the same susceptibility to NFAP as the R153 strain, and significant differences between the growth percentages at different NFAP concentrations were observed (*P* < 0.0001) (Table 1). These results suggest that NFAP has no influence on the PkcA signalling, which affects polarity establishment and induces apoptosis suppression (Katayama et al., 2012). Further evidence was gained by using a MpkA deletion mutant. MpkA is involved in the polarized growth of *A. nidulans* and the cell wall integrity (CWI) signalling pathway (Bussink and Osmani, 1999; Fujioka et al., 2007). Cell wall stressing agents (such as caffeine and CFW) induce the CWI pathway by increasing the Pkc and Mpk phosphorylation in *A. nidulans* (Fujioka et al., 2007). Indeed, we could show that the *alcA*-PkcA and *∆mpkA* strains were hypersensitive to caffeine and CFW compared to their isogenic control strains R153 and GR5 (Table 3). The *∆mpkA* strain, however, exhibited resistance to NFAP, while the recipient GR5 strain was susceptible (Table 1). This observation suggests that NFAP does not interfere directly with the activation of MpkA, but may affect a CWI pathway-independent MpkA-activated unknown target playing a role in the antifungal effect of NFAP.

**Table 3:** Growth percentages of *Aspergillus nidulans* strains in presence of caffeine and CFW in *in vitro* broth microdilution test after 48 h of incubation at 30°C or 37°C (depending on the investigated strain)

Compounds/strain	Caffeine		CFW		Type
	10 mmol/L	20 mmol/L	10 µg/mL	20 µg/mL	
RhoA^G14V^	78% ± 1.2%***	60% ± 8.4%*	92% ± 7.1%^ns^	55% ± 7.7%^ns^	Mutant
*∆mpkA*	17% ± 3.9%***	15% ± 2.6%***	76% ± 2.5%***	49% ± 4.0%**	Mutant
GR5	59% ± 1.1%	48% ± 2.7%	97% ± 2.1%	60% ± 3.3%	Isogenic recipient of RhoAG^14V^ and *∆mpkA*
*alcA*-PkcA	29% ± 0.3%***	21% ± 4.4%***	44% ± 5.9%***	22% ± 5.2%***	Mutant
R153	87% ± 2.9%	79% ± 14.4%	95% ± 3.0%	74% ± 2.6%	Isogenic recipient of *alcA*-Pkca

The untreated control is taken as 100% of growth. The mean growth percentages and their standard deviations from three replicates (*n* = 3) are indicated in the cells. Significant differences (*P*-values) were determined based on the comparison with the growth percentages of parental strain. ***: *P* < 0.0001; **: *P* < 0.005; *: *P* < 0.05, ^ns^: no significant differences. CFW: calcofluor white

#### NFAP does not activate cell wall integrity pathway

Based on our results we hypothesized that NFAP does not activate the CWI pathway by activation of downstream targets. To prove this assumption we investigated the sensitivity of *A. nidulans* FGSC A4 strain towards 100 µg/mL CFW, and 100 µg/mL CFW in combination with 200 µg/mL NFAP. CFW induces the CWI pathway by activation of the Rho/Pkc/Mpk signalling cascade in *A. nidulans* (Fujioka et al., 2007). In the presence of CFW and NFAP alone, this strain showed 9% ± 0.3% and 52% ± 3.2% growth, respectively; while significant increase in the growth was detected in combination of the two compounds (61.3% ± 1.3%, *P* < 0.0001). This ameliorated growth effect indicates that CFW and NFAP antagonize each other, and NFAP does not activate the CWI pathway; however, further experiments (e.g. investigation of CWI gene expression) are needed for verification of this assumption.

## DISCUSSION

From the 1990s several extracellular cysteine-rich proteins with remarkable antifungal activity against moulds have been isolated and characterized from filamentous Ascomycetes. The antifungal mode of action is extensively studied only in the case of three representatives, the *Aspergillus giganteus* antifungal protein (AFP) (Meyer, 2008), the *A. giganteus* A3274 antifungal protein (AFP_NN5353_ a very similar protein to AFP) (Binder et al., 2011) and the *Penicillium chrysogenum* antifungal protein (PAF) (Hegedus et al., 2011). Results presented in this study allow us to compare the detailed antifungal mechanism of NFAP with these proteins. By studying an NFAP-producing *A. nidulans* strain we previously proved that NFAP interferes with the organization of the cell wall, destroys the chitin filaments as it was observed also in the case of AFP, and triggers apoptotic-necrotic events through ROS accumulation as described for PAF (Galgóczy et al., 2013). However, it was not clarified whether these changes are the primary, short-time effect of the NFAP or if they are the consequences of its secondary, cell-killing effect.

NFAP caused metabolic inactivation and induced apoptotic cell death in *A. nidulans*, but no plasma membrane disruption could be observed within one hour of exposure. This observation is comparable with our previous finding when majority of the NFAP-producing *A. nidulans* germlings showed apoptotic phenotype after 8 h of cultivation suggesting the significance of apoptosis-inducing effect for antifungal activity (Galgóczy et al., 2013). Previously, it was proved that PAF evokes metabolic inactivity in *Aspergillus niger* (Kaiserer et al., 2003), and a PCD-like phenotype in *A. nidulans* (Leiter et al., 2005). Plasma membrane disruption by AFP was detected in *A. niger* (Theis et al., 2003).

Similar to our investigations with NFAP, disordered actin patch distribution, reduced chitin content and delocalized chitin deposition at the hyphal tips were also observed previously when *A. nidulans* was treated with PAF (Binder et al., 2010). During the polarized growth of *A. nidulans* exocytic vesicles (containing cell wall biosynthetic enzymes and wall precursors) and endocytic recycling vesicles are delivered by myosin molecules on actin cables to and from the hyphal tip. Therefore actin functions in the spatially coupled exo- and endocytosis at the tip and in holding the tip apparatus together (Taheri-Talesh et al., 2008). Since the correct localization of actin filaments is essential for polarized growth, the random distribution of actin as a consequence of NFAP treatment results in disturbed apical growth which leads to the previously well-described hyperbranching, swollen hyphal tips and destructed cell wall of the sensitive fungus in the presence of NFAP (Kovács et al., 2011; Galgóczy et al., 2013) (Fig. S3).

The indirect immunofluorescence staining experiments revealed that NFAP is internalized by passive transport across the disrupted sites of the plasma membrane and cell wall, not by an endocytotic uptake. The membrane disruption at hyphal fractures and bubbles where NFAP is accumulated after 16 h of treatment were confirmed by PI staining. These sites showed intensive red fluorescence indicating the loss of membrane integrity after more than one hour of incubation with NFAP (Fig. 2B). In contrast to NFAP, PAF and AFP_NN5353_ are internalized by endocytosis and distributed in the cytoplasm of *A. nidulans* (Oberparleiter et al., 2003; Binder et al., 2011), while AFP accumulates only in the outer layer, the cell wall and/or the plasma membrane of *A. niger* (Theis et al., 2003, 2005).

Based on the above mentioned observations NFAP may get into the cell within 30 min in a concentration below the detectable level which might be enough to interfere with the actin localization and chitin distribution and to cause apoptosis. It could be also possible that these effects are secondary consequences of the NFAP action: NFAP may bind to a surface receptor which activates a signalling pathway that leads to apoptosis and defective polar growth. The susceptibility tests with *A. nidulans* signalling mutants indeed point towards signalling pathways affected by NFAP.

**Figure 3:**
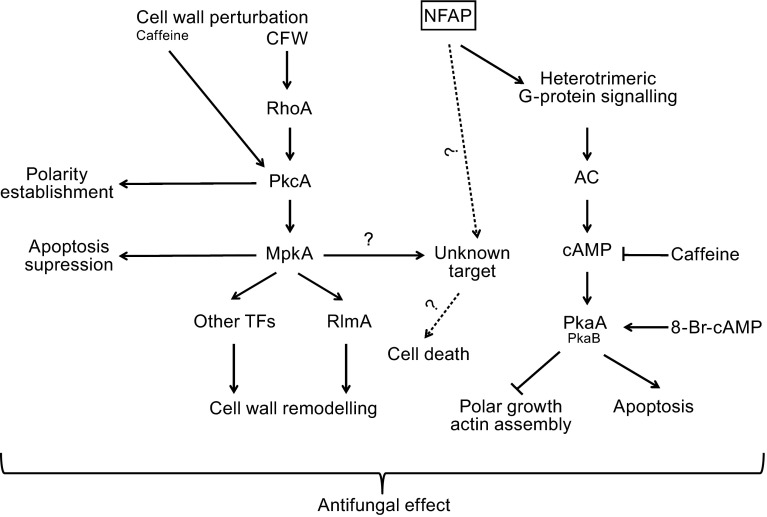
**Tentative model for the antifungal mechanism of** ***Neosartorya fischeri*** **NFAP** **in** ***Aspergillus nidulans*** **modified from Binder et al.** **(** 2010; 2011 **) for** ***Penicillium chrysogenum*** **PAF and** ***Aspergillus giganteus*** **A3274 AFP**_**NN5353**_ **, respectively**. 8-Br-cAMP: 8-bromoadenosine 3’,5’-cyclic monophosphate, AC: adenylate cyclase, cAMP: cyclic adenosine monophosphate, CFW: calcofluor white, Mpk: mitogen activated protein kinase, Pka: protein kinase A, Pkc: protein kinase C, RhoA: small GTP binding protein, RlmA: transcription factor, TFs: transcription factors

The antifungal mechanism of NFAP proved to be independent from the RhoA and PkcA, members of the CWI pathway. The small GTPase RhoA is an essential protein involved in the polar growth (Guest et al., 2004). PkcA plays a role in the polarity establishment independently of MpkA (Katayama et al., 2012) and in the suppression of apoptosis via MpkA (Katayama et al., 2012) (Fig. 3). Similarly, the toxicity of the *P.chrysogenum* antifungal protein PAF was also shown to be independent from RhoA, instead inhibition of RhoA-GAP targets was supposed (Binder et al., 2010). In contrast to NFAP, PAF possibly inactivates the Pkc signalling (Binder et al., 2010). Binder et al. (2011) suggested that the toxicity of AFP_NN5353_ is transmitted by RhoA-GAP targets and not by RhoA itself. The role of RhoA-GAP targets in the NFAP toxicity is awaiting further investigations. In our study NFAP did not induce the CWI pathway in *A. nidulans* similar to PAF (Binder et al., 2010). In contrast, AFP and AFP_NN5353_ activates the CWI pathway by increasing the MpkA/RlmA-activated α-glucan synthase *agsA* expression, a main enzyme in the cell wall remodelling of *A. niger* (Hagen et al., 2007; Binder et al., 2011) (Fig. 3).

Our results indicate that NFAP might bind to a G-protein coupled receptor similarly to other antifungal proteins and drugs (Van Dijck, 2009; Hegedüs and Marx, 2013) that results in the induction of the cAMP/Pka cascade, which inhibits the polar growth (Bencina et al., 2005) and modulates PCD (Semighini et al., 2006) (Fig. 3). Similar to NFAP, PAF also activates the cAMP/Pka signalling cascade via the heterotrimeric G-protein signal transduction pathway (Binder et al., 2010). The identification of a G-protein coupled receptor of NFAP would further accelerate the elucidation of the mechanism of its antifungal activity.

Presumably, activation of a cell wall integrity pathway-independent, but MpkA-dependent unknown factor, which induces PCD, may also play a role in the antifungal mechanism of NFAP (Fig. 3). This latter assumption is supported by the report of the existence of an MpkA-mediated cell death pathway in *A. nidulans* by Colabardini et al. (2010). This group observed an increased tolerance of the *A. nidulans∆mpkA* strain to farnesol-induced cell death compared to *∆mpkC* and *∆hogA* strains. Proving the existence of this MpkA-activated apoptosis factor is waiting for further investigations. In contrast to NFAP, PAF fails to activate the Mpk (Binder et al., 2010), and AFP_NN5353_ evokes growth inhibition via activation of Pkc/Mpk signalling pathway in *A. nidulans* (Binder et al., 2011).

The features of the cysteine-rich antifungal proteins render them exceptionally suitable compounds as commercial preservatives, bio-pesticides and drugs against moulds and offer an alternative, safely applicable solution for the recent antifungal challenges (Marx et al., 2008; Meyer, 2008). Our present results significantly contribute to the understanding of the antifungal mechanism not only of NFAP, but also of other cysteine-rich antifungal proteins from Ascomycetes in general.

## MATERIALS AND METHODS

### Strains and media

*A. nidulans* strains used in the present study are listed in Table 4. All signalling mutants, their isogenic recipients, and the actin-GFP strain were grown and maintained in minimal medium (MM, Ronen et al., 2007) with respective supplementation (Bussink and Osmani, 1999; Shimizu and Keller, 2001; Guest et al., 2004, Ronen et al., 2007; Taheri-Talesh et al., 2008). The Glasgow wild type *A. nidulans* FGSC 4A was grown and maintained in complete medium (CM, Kuranda et al., 2006).

**Table 4:** Investigated *Aspergillus nidulans* strains and their genotype

Strain	Relevant genotype	Reference or source
FGSC A4	Glasgow wild type	FGSC
FGSC 116	*yA2*	FGSC
FGSC 1035	*yA2 fadAG203R*	FGSC
R153	*wA2; pyroA4*	Ronen et al. (2007)
*alcA*-PkcA	*wA2; pyroA4; pyrG89*::*pyr4alcA*(*p*)::*pkcAΔp*	Ronen et al. (2007)
GR5	*pyrG89; wA3; pyroA4*	Guest et al. (2004)
RhoA^G14V^	*A773* *+* *pGG2* (*rhoA* ^*G14*^) and pRG3AMA1 (co-transformation plasmid)	Guest et al. (2004)
*ΔmpkA*	*ΔmpkA*	Bussink and Osmani (1999)
RKIS1	*papaA1; yA2*	Shimizu and Keller (2001)
*ΔpkaA*	*papaA1; yA2; ΔpkaA*::*argB; ΔargB*::*trpC; trpC801; veA1*	Shimizu and Keller (2001)
Actin-GFP	*wA3; pyroA4; actin_GFP*::*pyrG* (*pyr G 89*)	Taheri-Talesh et al. (2008)

FGSC: Fungal Genetics Stock Center, Kansas, MO, USA

### NFAP production and purification

Heterologous expression and purification of NFAP was carried out in *P. pastoris* KM71H as described previously (Virágh et al., 2014).

### Microscopic investigation

Microscopic investigation of the NFAP-specific antifungal effect was carried out on *A. nidulans* FGSC A4 and *A. nidulans* actin-GFP conidia (10^5^ conidia/mL) which were immobilised on cover slips, and incubated in CM at 37°C (or at 30°C in case of *A. nidulans* actin-GFP strain) for 16 h. For the investigation of the short-time antifungal effect, cover slips were treated with CM supplemented with sublethal concentration of NFAP (25 µg/mL) for 30 and 60 min at 37°C (or at 30°C in case of *A. nidulans* actin-GFP strain). CM without NFAP was used as control. To reveal the long-time antifungal effect cover slips were incubated in the presence of NFAP for 16 h at 37°C (or at 30°C in case of *A. nidulans* actin-GFP strain).

Germinated conidia and/or hyphae were examined and photographed with a light microscope with fluorescence lamp (LR 66238C, Carl Zeiss, Axiolab) equipped with a digital microscope camera (AxioCam ERc 5s, Carl Zeiss, Axiolab). All experiments were repeated three times.

### Viability staining and detection of apoptotic/necrotic events

To compare the metabolic activity of NFAP-treated and untreated *A. nidulans* FGSC A4 hyphae and to reveal the apoptotic/necrotic events, FUN1 viability staining (Invitrogen-Life Technologies, Eugene, OR, USA) and Annexin V-FITC Apoptosis Detection Kit (Sigma-Aldrich, St. Louis, MO, USA) were adapted to cover slips, respectively. After incubation, the hyphae on cover slips were washed with 10 mmol/L HEPES pH = 7.5 (Sigma-Aldrich, St. Louis, MO, USA), then incubated for 30 min at 37°C in 10 mmol/L HEPES containing 2% (*w*/*v*) glucose (pH = 7.5), and stained with 5 µmol/L FUN1 stain diluted in 10 mmol/L HEPES pH = 7.5 in the dark at room temperature for 30 min, and finally, washed again with 10 mmol/L HEPES supplemented with 2% (*w*/*v*) glucose (pH = 7.5). In case of the Annexin V-FITC Apoptosis Detection Kit the hyphae on cover slips were washed with CM and stained with PI and Annexin V-FITC diluted in binding buffer in the dark at room temperature for 10 min, and then washed again with CM.

### Detection of membrane disruption

PI staining was used to observe NFAP-specific membrane disruption on *A. nidulans* FGSC A4. Cover slips treated with 70% (*v*/*v*) ethanol for 30 min at 4°C were used as positive staining controls. After washing with CM cover slips were stained with 5 µg/mL PI for 10 min at room temperature in the dark, and then washed again with CM.

### Analysis of actin distribution and chitin content

To investigate the impact of NFAP on the actin distribution a derivate of the *A. nidulans* GR5 strain was used, which carries an additional copy of actin tagged with green fluorescent protein on its N-terminus (Taheri-Talesh et al., 2008). Chitin content of the *A. nidulans* FGSC A4 in the presence of NFAP was visualized with CFW staining (Sigma-Aldrich, St. Louis, MO, USA). Cover slips were washed with CM, then stained with 10 µmol/L CFW at room temperature in the dark for 10 min, and finally, washed again with CM.

### Generation and testing of anti-NFAP polyclonal sera

Two-months-old female WISTAR rats were immunized with 100 μg NFAP in complete, then twice in incomplete Freund’s adjuvant. The animal experiments we performed according to institutional and national ethical guidelines, in possession of ethical clearances. Individual sera from three rats were screened by Western blotting, and the serum with the strongest specific signal was used in further experiments. For the screening of the sera, purified NFAP was separated by SDS-PAGE (XCell SureLock Mini-Cell, Invitrogen-Life Technologies, Eugene, OR, USA) and transferred onto PVDF membranes (Immobilon-P, IPVH00010, Millipore, Billerica, MA, USA). Membrane was incubated overnight at 4°C with NFAP-immunized rat serum (1:100), washed three times, and then incubated with horseradish peroxidase conjugated anti-rat secondary antibody (1:1000; R&D, Minneapolis, MN, USA). Immunoreactive signals were developed using Supersignal West Pico Chemiluminscent Substrates (Thermo Scientific, MR, USA) and detected with LI-COR ODYSSEY® Fc (Dual-mode imaging system) imager followed by analysis with Odyssey v1.2 software.

### Immunofluorescence staining

Localization of NFAP in *A. nidulans* FGSC A4 was investigated using an indirect immunofluorescence staining method described by Fischer and Timberlake (1995). Cover slips with germlings were transferred to a solution containing 50 mmol/L PIPES (pH = 6.7), 25 mmol/L EGTA, 5 mmol/L MgSO_4_, 5% (*v*/*v*) dimethyl sulfoxide, 8% (*v*/*v*) formaldehyde. After this fixation step, cover slips were washed four times with PEM buffer (50 mmol/L PIPES pH = 6.7, 25 mmol/L EGTA, 5 mmol/L MgSO_4_). For wall digestion coverslips were transferred to PEM buffer containing Glucanex (500 mg/mL; Sigma-Aldrich, St. Louis, MO, USA) and incubated at room temperature for 2 h, then washed four times in PEM buffer (pH = 6.7) and transferred into an extraction solution containing 100 mmol/L PIPES (pH = 6.7), 20 mmol/L EGTA, 0.1% (*v*/*v*) Triton X-100. After this treatment the cover slips were incubated in the presence of rat anti-NFAP serum diluted 1:600 in Tris-buffered saline containing bovine serum albumin (TBS/B, 20 mmol/L Tris-HCl pH = 8.0, 20 mmol/L NaCl, 0.1% (*v*/*v*) Tween-20, 3% (*v*/*v*) bovine serum albumin (Sigma-Aldrich, St. Louis, MO, USA)) for 60 min. Immunocomplexes were detected with FITC-conjugated goat-anti-rat-IgG (Sigma-Aldrich, St. Louis, MO, USA) diluted 1:40 in TBS/B. For studying the uptake mechanism of NFAP, immunofluorescence staining was performed after treatment of the samples with NFAP and 5 µg/mL latB (inhibitor of actin polymerization; Sigma-Aldrich, St. Louis, MO, USA). As a positive control, indirect immunofluorescence staining was repeated with monoclonal mouse anti-actin antibody (Sigma-Aldrich, St. Louis, MO, USA) and polyclonal rabbit anti-mouse immunoglobulins/TRITC (Agilent Technologies, Dako Denmark A/S, Glostrup, Denmark).

### Growth inhibition assay

The antifungal effect of NFAP (25–200 µg/mL), 8-Br-cAMP (5 mmol/L), CFW (10, 20, and 100 µg/mL), and caffeine (10 and 20 mmol/L) (all from Sigma-Aldrich, St. Louis, MO, USA) was investigated in a 96-well microtiter plate bioassay on *A. nidulans* strains in CM or MM containing 10^5^ conidia/mL and supplemented with the required ingredients for the growth of certain strains (Bussink and Osmani, 1999; Shimizu and Keller, 2001; Guest et al., 2004, Ronen et al., 2007). In the combination experiments 100 μL aliquots of a serial NFAP dilution (50–200 µg/mL) containing 2 × 10^5^ conidia/mL were mixed with 100 μL 100 µg/mL CFW, 5 mmol/L 8-Br-cAMP, or 20 mmol/L caffeine at final concentrations in the wells. Plates were incubated at the optimal temperature of the tested strain for 48 h without shaking and then the absorbances (OD_620_) were measured after gently shaking for two seconds with a microtiter plate reader in well scanning mode (SPECTROstar Nano, BMG Labtech, Ortenberg, Germany) (Tables S1, S2, and S3). Fresh medium was used for background calibration. For calculation of the growth rates, the absorbances of the untreated control cultures were set to be 100% growth. All susceptibility tests were repeated three times.

### Statistical analysis

All statistical analyses were performed using GraphPad Prism version 5.01 for Windows (GraphPad Software, San Diego, CA, USA). The significant differences between sets of data were determined by One-way analysis of variance with Bonferroni’s multiple comparison posttest according to the data.

## Electronic supplementary material

Supplementary material 1 (PDF 235 kb)
