# Supplementary material for: Insight into the antifungal mechanism of Neosartorya fischeri antifungal protein
Source: Protein Cell. 2015 May 22;6(7):518–28. doi: 10.1007/s13238-015-0167-z (PMC4491047; doi:10.1007/s13238-015-0167-z)
Supplement: Supplementary file 1 — Supplementary material 1 (PDF 235 kb) [file 13238_2015_167_MOESM1_ESM.pdf]

Supplementary material

Article title: Insight into the antifungal mechanism of *Neosartorya fischeri* antifungal protein

Author names: Máté Virágh, Annamária Marton, Csaba Vizler, Liliána Tóth, Csaba Vágvölgyi, Florentine Marx, László Galgóczy

Journal name: Protein & Cell

Affiliation and email address of the corresponding author: Department of Microbiology, Faculty of Science and Informatics, University of Szeged, Szeged, Hungary; galgoczi@gmail.com

Table S1. Measured OD_{620} values for the calculation of the growth percentages of the investigated *Aspergillus nidulans* strains in presence of different concentrations of NFAP in *in vitro* broth microdilution test after 48 hours of incubation at 30 or 37 °C (depending on the investigated strain).

NFAP / Strain	0 μg ml^{-1}	200 μg ml^{-1}	100 μg ml^{-1}	50 μg ml^{-1}	Type
RhoA^{G14V}	0.741±0.081	0.354±0.112	0.420±0.033	0.637±0.093	mutant
Δ*mpkA*	0.554±0.037	0.598±0.050	0.584±0.037	0.572±0.005	mutant
GR5	1.466±0.432	0.686±0.110	0.856±0.079	1.216±0.175	isogenic recipient of RhoAG^{14V} and Δ*mpkA*
alcA-PkcA[a]	0.810±0.205	0.385±0.086	0.467±0.044	0.518±0.102	mutant
alcA-PkcA[b]	0.436±0,058	0.321±0,033	0.336±0,002	0.433±0.001	mutant
R153	0.796±0.042	0.573±0.023	0.661±0.035	0.819±0.015	isogenic recipient of *alcA*-Pkca
Δ*pkaA*	0.939±0.206	0.530±0.057	0.648±0.050	0.783±0.108	mutant
RKIS 1	0.906±0.227	0.364±0.025	0.436±0.015	0.602±0.037	isogenic recipient of Δ*pkaA*
FGSC 1035	0.800±0.091	0.414±0.016	0.624±0.004	0.810±0.004	mutant
FGSC 116	0.796±0.141	0.338±0.053	0.473±0.015	0.596±0.018	isogenic recipient of FGSC 1035

The mean OD_{620} values and their standard deviations from three replicates (N=3) are indicated in the cells. [a]: *A. nidulans alcA*-PkcA in the presence of glucose, [b]: *A. nidulans alcA*-PkcA in the presence of glycerol.

Table S2. Measured OD_{620} values for the calculation of growth percentages of *Aspergillus nidulans* strains in presence of NFAP and NFAP - 8-Br-cAMP/caffeine combinations in *in vitro* broth microdilution test after 48 hours of incubation at 37 °C.

NFAP / Other compounds	0 µg ml^{-1}	50 µg ml^{-1}	100 µg ml^{-1}	200 µg ml^{-1}
FGSC A4				
NFAP	1.633±0.077	1.480±0.098	1.087±0.109	0.853±0.052
NFAP + 5 mM 8-Br-cAMP	1.149±0.027	0.948±0.068	0.931±0.062	0.667±0.053
NFAP + 20 mM caffeine	1.152±0.035	1.207±0.054	0.999±0.115	1.761±0.025
ΔpkaA				
NFAP	0.939±0.206	0.783±0.108	0.648±0.050	0.530±0.057
NFAP + 20 mM caffeine	0.593±0.061	1.150±0.074	1.826±0.287	1.842±0.029

The mean OD_{620} values and their standard deviations from three replicates (N=3) are indicated in the cells.

Table S3. Measured OD_{620} values for the calculation of growth percentages of *Aspergillus nidulans* strains in presence of caffeine and CFW in *in vitro* broth microdilution test after 48 hours of incubation at 30 or 37 °C (depending on the investigated strain).

Compounds/Strain	Caffeine		CFW		Type
	10 mM	20 mM	10 µg ml^{-1}	20 µg ml^{-1}	
RhoA^{G14V}	0.579±0.011	0.441±0.062	0.679±0.053	0.407±0.057	mutant
Δ*mpkA*	0.095±0.022	0.084±0.013	0.422±0.014	0.272±0.022	mutant
GR5	0.871±0.018	0.705±0.040	1.425±0.031	0.881±0.048	isogenic recipient of RhoAG^{14V} and Δ*mpkA*
alcA-PkcA	0.463±0.005	0.343±0.071	0.710±0.095	0.361±0.084	mutant
R153	0.692±0.023	0.630±0.115	0.757±0.024	0.591±0.021	isogenic recipient of *alcA*-Pkca

The mean OD_{620} values and their standard deviations from three replicates (N=3) are indicated in the cells.

(a)

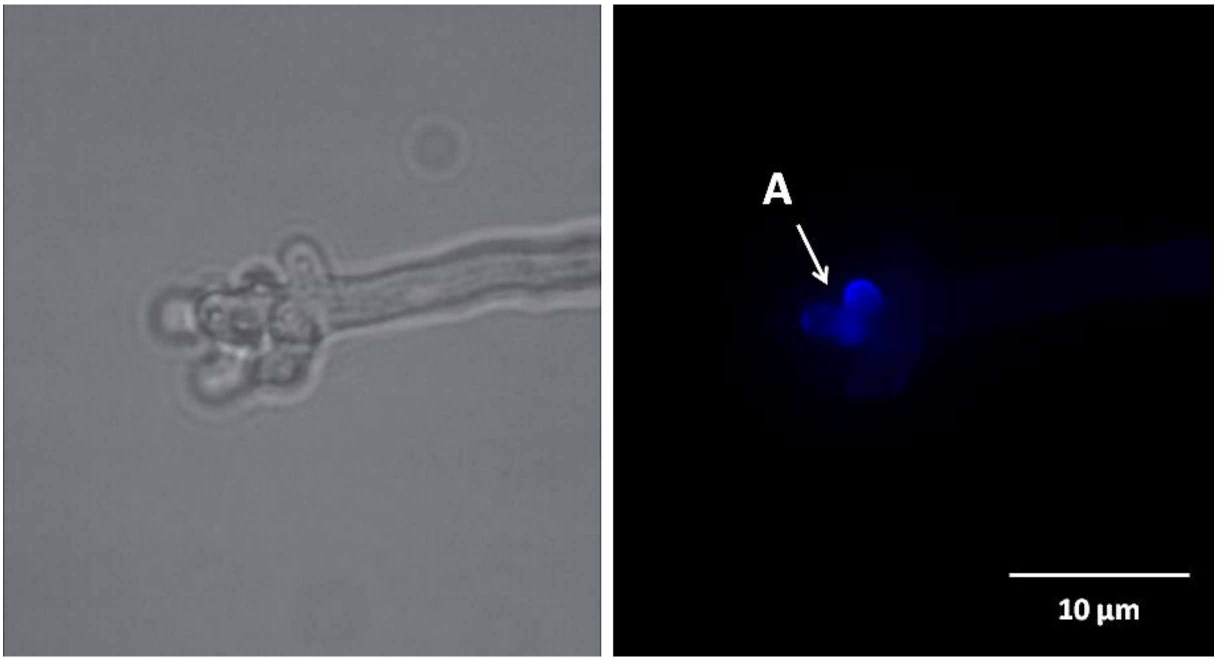

(b)

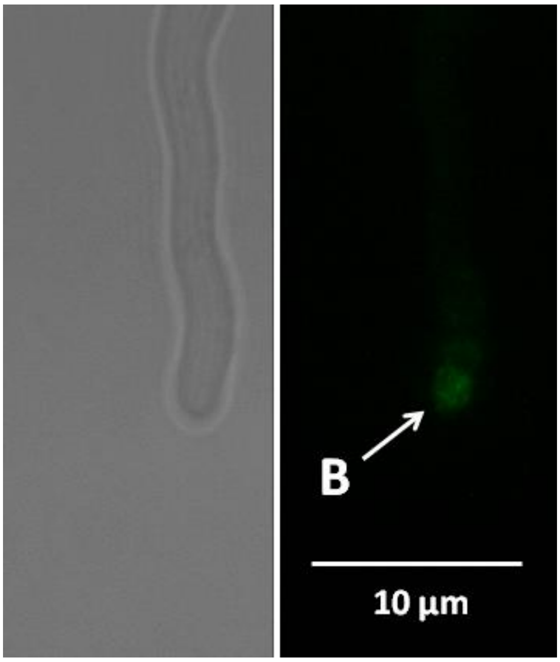

Fig S1. Calcofluor white (CFW) staining of *Aspergillus nidulans* FGSC A4 hyphae after 8-Br-cAMP treatment for 60 minutes at 37 °C (a). Actin distribution at *Aspergillus nidulans* Actin-GFP hyphal tips in response to 8-Br-cAMP treatment for 60 minutes at 30 °C (b). A: delocalized chitin deposition and lack of the cap-like CFW fluorescence, B: abnormal actin deposition.

(a)

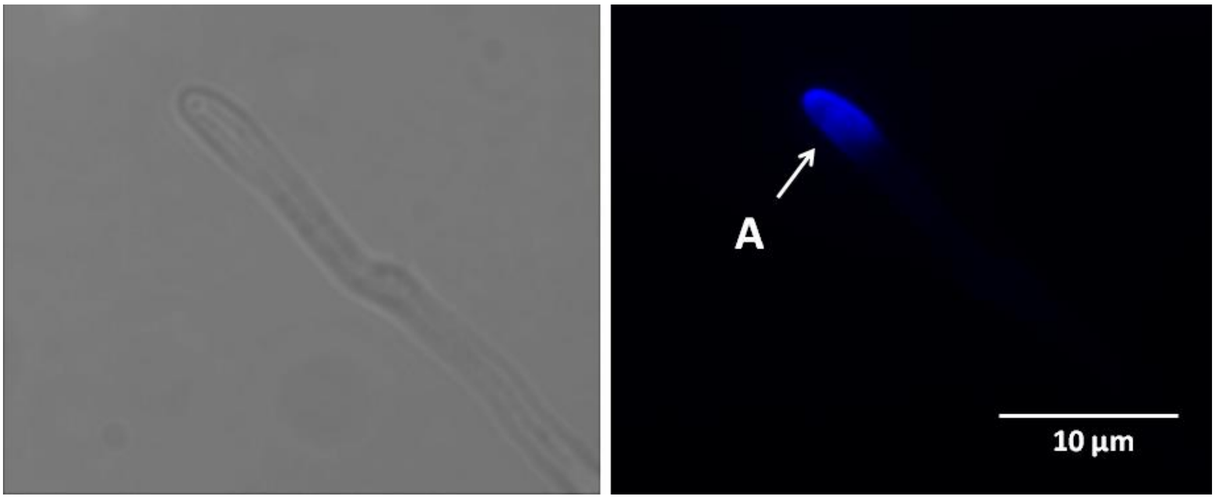

(b)

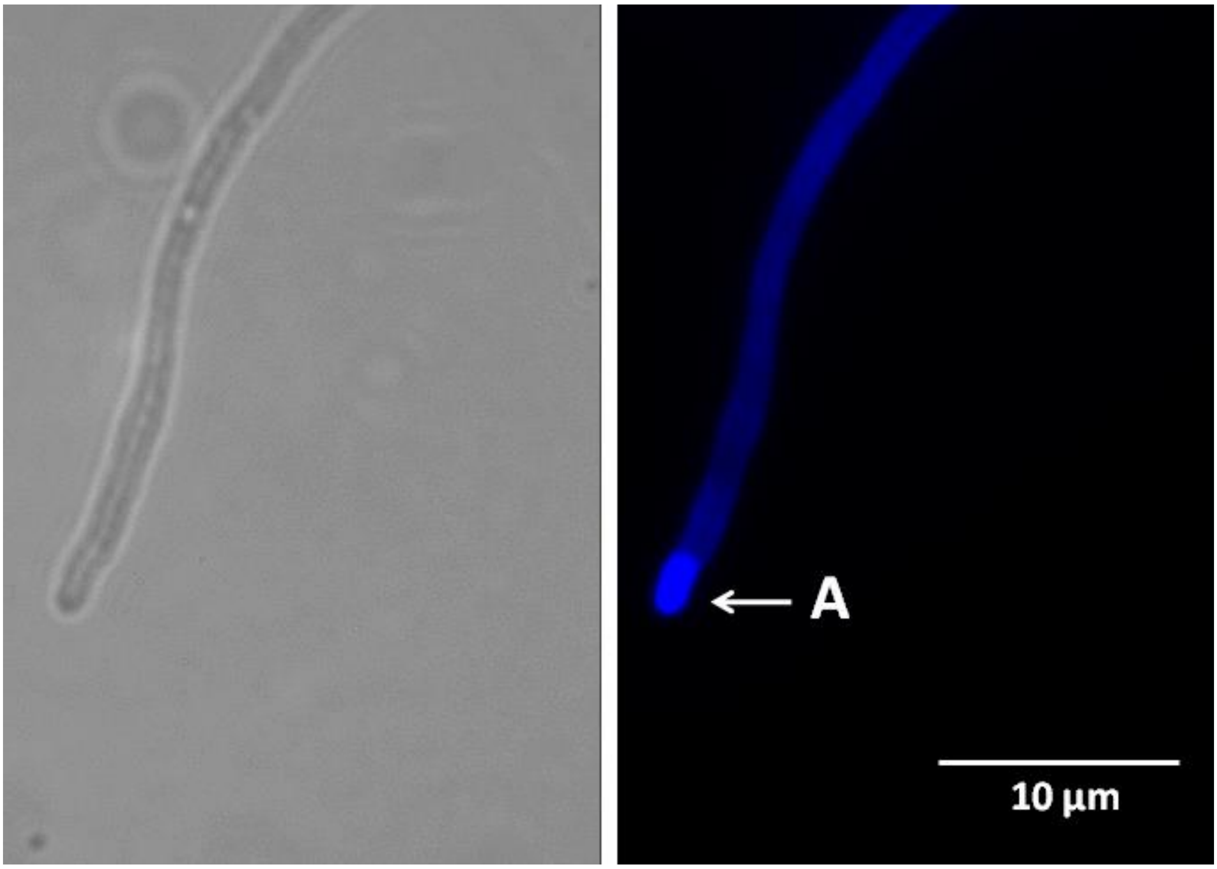

Fig S2. Calcofluor white (CFW) staining of *Aspergillus nidulans* FGSC 1035 (a) and Δ*pkaA* (b) hyphae after 25 µg ml^{-1} NFAP treatment for 30 minutes at 37 °C. A: cap-like CFW fluorescence - site of the chitin assembly.

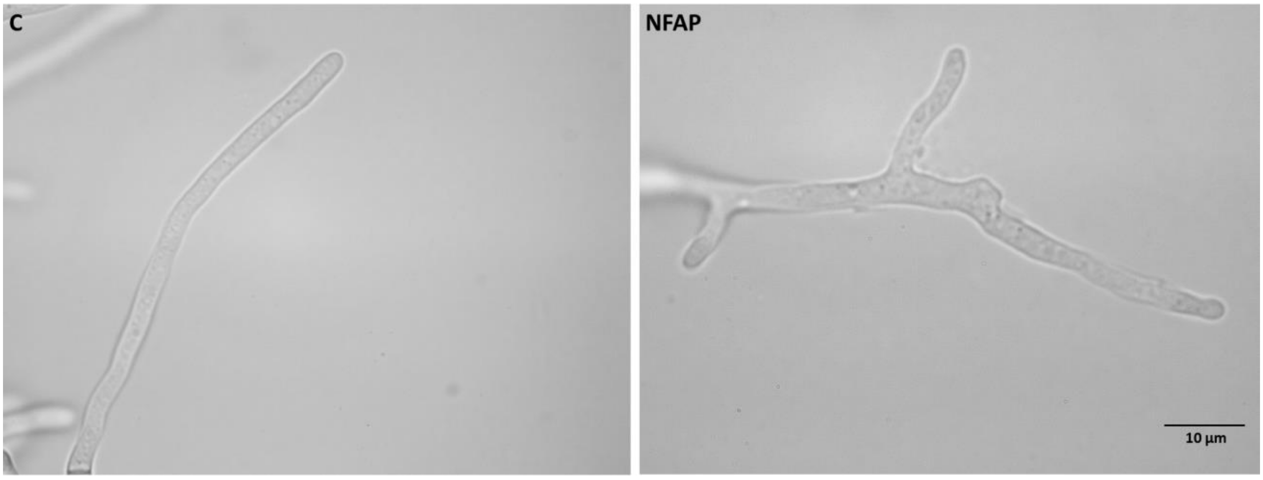

Fig S3. Morphology of *Aspergillus nidulans* FGSC A4 hyphae after incubation at 37 °C for 20 hours in complete medium supplemented with sublethal concentration of NFAP. C: untreated control, NFAP: NFAP-treated (25 µg ml^{-1}) hyphae.
